# Effects of eccentric exercise in patients with subacromial impingement syndrome: a systematic review and meta-analysis

**DOI:** 10.1186/s12891-019-2796-5

**Published:** 2019-10-14

**Authors:** Robin Larsson, Susanne Bernhardsson, Lena Nordeman

**Affiliations:** 1 Capio Rehab Angered, Gothenburg, Sweden; 2 The Sahlgrenska Academy, Institute of Neuroscience and Physiology, Department of Health and Rehabilitation, Unit of Physiotherapy, University of Gothenburg, Gothenburg, Sweden; 3 Region Västra Götaland, Research and Development Primary Health Care, Gothenburg, Sweden; 4 Region Västra Götaland, Research and Development Primary Health Care, Borås, Sweden

**Keywords:** Subacromial impingement syndrome, shoulder impingement syndrome, Subacromial pain syndrome, Eccentric exercise, Eccentric training

## Abstract

**Background:**

Subacromial impingement syndrome is a common problem in primary healthcare. It often include tendinopathy. While exercise therapy is effective for this condition, it is not clear which type of exercise is the most effective. Eccentric exercises has proven effective for treating similar tendinopathies in the lower extremities. The aim of this systematic review was therefore to investigate the effects of eccentric exercise on pain and function in patients with subacromial impingement syndrome compared with other exercise regimens or interventions. A secondary aim was to describe the included components of the various eccentric exercise regimens that have been studied.

**Methods:**

Systematic searches of PubMed, Cochrane Library and PEDro by two independent authors. Included studies were assessed using the PEDro scale for quality and the Cochrane scale for clinical relevance by two independent authors. Data were combined in meta-analyses. GRADE was applied to assess the certainty of evidence.

**Results:**

Sixty-eight records were identified. Seven studies (eight articles) were included, six were meta-analysed (*n* = 281). Included studies were of moderate quality (median PEDro score 7, range 5–8). Post-treatment pain was significantly lower after eccentric exercise compared with other exercise: MD -12.3 (95% CI − 17.8 to − 6.8, I^2^ = 7%, *p* < 0.001), but this difference was not clinically important. Eccentric exercise provided no significant post-treatment improvement in function compared with other exercise: SMD -0.10 (95% CI − 0.79 to 0.58, I^2^ = 85%, *p* = 0.76). Painful eccentric exercise showed no significant difference compared to pain-free eccentric exercise. Eccentric training regimes showed both similarities and diversity. Intervention duration of 6–8 weeks was almost as effective as 12 weeks.

**Conclusions:**

Evidence of low certainty suggests that eccentric exercise may provide a small but likely not clinically important reduction in pain compared with other types of exercise in patients with subacromial impingement syndrome. It is uncertain whether eccentric exercise improves function more than other types of exercise (very low certainty of evidence). Methodological limitations of existing studies make these findings susceptible to change in the future.

**Trial registration:**

PROSPERO CRD42019126917, date of registration: 29-03-2019.

## Background

Subacromial impingement syndrome is a common healthcare problem, especially in adult populations. Prevalence is estimated to between seven and 26% of the general population [1], and almost half of all shoulder-related pain in patients seeking primary health care is related to subacromial impingement syndrome [2]. A thorough understanding of the treatment of this condition is therefore of importance for physiotherapists and other healthcare personnel.

Subacromial impingement syndrome is a condition where the subacromial space, the area directly below the acromion process and above the shoulder joint, has narrowed. This can happen for several reasons, traditionally categorised as either primary or secondary causes. Primary causes are structural changes or morphological pre-conditions of the acromion process [3, 4]. Secondary causes often depend on multiple factors such as rotator cuff syndrome (strains or tears to the muscles and/or tendons of the muscles making up the rotator cuff), tendinopathy (inflammation or degeneration) in one or more of the same muscle tendons (infraspinatus, supraspinatus or subscapularis) and/or inflammation of the subacromial bursa [5, 6]. The long head/tendon of the biceps brachii can also be involved [6].

Eccentric exercises are exercises performed only during the elongation phase of muscle activation (i.e. the lowering or slowing-down phase of a limb) and normally at a high intensity. A possible hypothesis for their benefits is that they could potentially reverse painful neovascularization within damaged tendons, which has been shown in a study on eccentric exercise and Achilles tendinopathy [7]. Eccentric exercises have also been shown to decrease swelling of the Achilles tendon [8].

Due to their high intensity, and the fact that collagen growth in tendons tends to peak 24 to 72 h after training [9], enough time for recovery seems vital for effective rehabilitation with eccentric exercises. It could therefore be expected that in eccentric exercise regimens, not only how the exercise is performed (eccentric versus concentric phase) and its intensity, but also its frequency and duration will be of importance.

It also has been proposed that injuries to tendons and other soft tissue are the most common causes of long-term shoulder pain in general. Histological examinations of tendon injuries of the supraspinatus in patients with subacromial impingement syndrome have shown changes to the tendon resembling those in patients with similar injuries of the patellar and Achilles tendon [10]. Patellar and Achilles tendinopathy are two types of tendon injuries where high intensity eccentric exercise has been shown effective, not only in decreasing pain but also in stimulating tissue regeneration and restoring function [11–13]. This makes it relevant to investigate whether eccentric exercises could be equally effective in treating patients with subacromial impingement syndrome.

Earlier studies have shown that exercise, in general, is effective in treating subacromial impingement syndrome [14], at least as effective as corticosteroid injections for treating pain [15] and equally long-term effective as surgery [16]. Eccentric exercise in particular also has shown promising results in three uncontrolled studies [17–19].

It is not clear at present which type of exercise/training is the most effective for subacromial impingement syndrome, and whether this differs depending on involved structures and underlying mechanisms [14, 16, 20, 21]. Previous reviews [22–24] on eccentric exercise and subacromial impingement syndrome have only had access to a limited set of data, up to two randomised controlled trials (RCTs) [25, 26] and one or more of the uncontrolled studies mentioned above [17–19]. A new review including recently published studies and incorporating a meta-analysis, not previously performed, would therefore be able to generate new knowledge, especially since this is a relatively new field of research [22]. Therefore, the aim of this systematic review was to investigate the effects of eccentric exercise on pain and function in patients with subacromial impingement syndrome compared with other exercise regimens or interventions. A secondary aim was to describe the included components of the various eccentric exercise regimens that have been studied.

## Methods

### Protocol and registration

A protocol for this systematic review was registered in PROSPERO (PROSPERO 29-03-2019: CRD42019126917). The review was conducted and reported according to the Preferred Reporting Items for Systematic Review and Meta-Analysis statement [27].

### Eligibility criteria

Participants: adult men and women, i.e. individuals from 18 years of age and upwards, with shoulder pain and diagnosed with subacromial impingement syndrome, defined as shoulder pain and a positive Neer’s impingement test, Hawkins-Kennedy impingement test, Empty can test (Jobe’s impingement test), Painful arc sign and/or positive response to a subacromial corticosteroid injection. Included individuals should be part of the general population and not only part of a specific subgroup, e.g. swimmers or tennis players, to allow for greater clinical relevance and applicability of the findings to the general population.

Intervention: eccentric exercise. The exercise interventions needed to be described in sufficient detail so that both exercise and execution could be clearly identified and so that the eccentric loading could be easily compared with any control, whether resistance training, other forms of eccentric exercise (e.g. pain versus no pain), or any other intervention. Eccentric exercises had to be the primary treatment method.

Comparisons: Other types of exercise (e.g. resistance, mobility, aerobic); other interventions (e.g. massage, mobilization/manipulation, acupuncture, TENS, corticosteroid injections); other types of eccentric exercise (where exercising according to different ratings of perceived pain have been compared).

Outcome measures: Pain, e.g. measured by visual analogue scale (VAS) or numerical pain rating scale (NPRS); function, e.g. measured by the disabilities of the arm, shoulder and hand (DASH) questionnaire or the Constant-Murley score; main components of the various eccentric exercise regimens.

Exclusion criteria were any uncontrolled study designs and shoulder pain due to fractures, dislocations or medical conditions (e.g. osteoarthritis, rheumatoid arthritis). Only RCTs qualified for inclusion because we aimed to assess intervention effects. Identification of any controlled but not randomised studies was reported. No limitation to population size was set.

### Literature search

We conducted literature searches in the databases PubMed, Cochrane Library and the Physiotherapy Evidence Database (PEDro) in March 2019. We developed a search strategy for PubMed and subsequently adapted it to the other databases (Appendix 1). The search strategy combined search terms with medical subject headings and comprised a combination of the term ”subacromial impingement”, or synonyms thereof, with the term ”eccentric exercise”, or synonyms thereof. We did not apply any restrictions to language or date of publication. We performed backward and forward citation searches of included studies and identified previous reviews for additional potentially relevant studies, and searched ClinicalTrials.gov for ongoing studies. We also consulted content experts in the field.

### Study selection

Two authors (RL and SB) screened the identified titles and abstracts independently for relevance (according to the inclusion/exclusion criteria) and assessed full text articles for inclusion. The authors were not blinded to trial identifiers such as authors’ and journals’ names.

### Data collection process

Two authors (RL and SB) performed data extraction independently. Extracted data included study aim, population demographics (including age and sex), intervention components, control group intervention components, outcome measures and outcome data. When needed, we solicited and obtained additional data from trial investigators.

### Risk of bias assessment

We assessed risk of bias in the included studies in duplicate (RL and SB or LN), using the PEDro scale for quality [28]. This instrument has been shown to have acceptably high reliability and validity [29, 30]. We also assessed clinical relevance using the Cochrane scale for clinical relevance [31]. Any disagreements were resolved by discussion among all authors until consensus was reached. To assess agreement among the reviewers, percentage agreement and Cohen’s kappa with 95% confidence intervals (CI) was calculated. The results of the risk of bias assessment was used, together with other criteria, in the overall assessment of the certainty of the evidence.

### Data synthesis and analysis

When possible, we combined the data in meta-analyses for investigation of the aggregated post-treatment and intermediate to long-term effects. Because one study [32] compared painful versus pain-free eccentric exercise, we excluded it from the meta-analysis. When articles reported several different measures for pain, we extracted and tabulated all measures, but we chose one for the meta-analyses. “Worst pain” or “pain during activity” was chosen because we considered this measure to best correspond to “pain” in the other included studies. We also considered this to be the outcome most important to the patient.

We analysed pain by calculating the MD with corresponding 95% CI. To be able to present the aggregated effect, we rescaled the NPRS data from 0 to 10 to 0–100. When median and percentiles were reported instead of mean and SD, we approximated the missing mean and SD with the corresponding median and percentile range and imputed these values [33]. We assumed a normal distribution of pain scores on the VAS or the NPRS, and we considered a 15 mm difference on the VAS as representative of a minimal important difference (MID) in pain [34].

For function, five different instruments were used in the seven studies. Due to the many instruments, we calculated the aggregated effect using standardised mean difference (SMD) with 95% CI. Because the scales went in different directions, mean values were multiplied by − 1 when necessary.

Statistical heterogeneity was assessed with the χ^2^ and I^2^ statistics. Because heterogeneity was present (I^2^ > 30%), we used random-effect models. When possible, missing data were handled by imputing values from previous time-points, applying the “last observation carried forward” principle. We analysed outcomes post-treatment in two subgroups which were not pre-planned: six to 8 weeks and 12 weeks. Intermediate and long-term outcomes were analysed when reported, as time points closest to 1 year. We explored whether excluding high risk of bias trials would affect the result. We performed the meta-analyses in Revman 5.3 [35].

### Assessment of certainty of evidence

To assess confidence in the combined estimates of effect, we applied the GRADE (Grading of Recommendations Assessment, Development and Evaluation) approach using the following criteria: risk of bias, consistency, directness, precision, and reporting bias [36]. Because all included studies were RCTs, we initially assigned a high certainty level, but rated down one or more levels to moderate, low or very low if we detected issues with risk of bias, precision, consistency or directness. Publication bias was not assessed due to the small number of studies, but was not considered likely.

## Results

### Search results

**Fig. 1:**
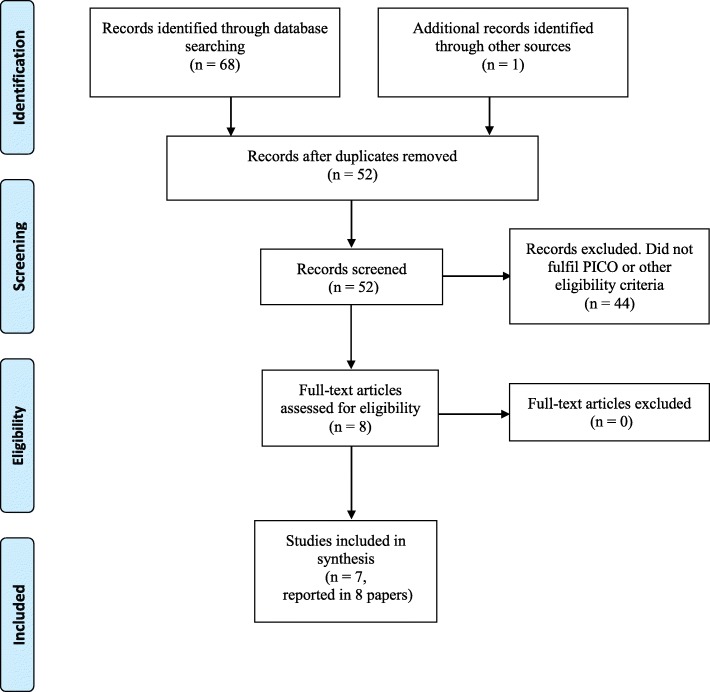
Flow diagram of the selection process. Modified from Moher et al., 2009

The search procedure yielded 68 records, of which 51 unique articles remained after removing duplicates. After screening titles and abstracts for relevance, and when necessary assessing the full text articles, 43 articles could be excluded according to inclusion/exclusion criteria. No controlled studies that were not also randomised were identified. Scrutiny of reference lists identified one potentially relevant study, but on closer inspection it did not fulfil the inclusion criteria. In total, we included seven studies reported in eight articles [25, 26, 32, 37–41]. The search process and results are illustrated in Fig. 1. Of the included studies, six were included in the meta-analyses [25, 26, 37–41].

### Characteristics of included studies

The seven included studies were conducted between 2012 and 2018. Two were conducted in the United States and five in Europe. Of the seven studies, six [25, 26, 37–40] compared eccentric exercise with other exercise. One [32] compared painful eccentric exercise (above the pain threshold: VAS < 40–50 mm) with pain-free eccentric exercise (VAS = 0 mm). For one of the studies, long-term results were reported in a separate article [41].

#### Risk of bias

Included studies had a PEDro score of between five and eight (out of 10), with a median value of seven (Table 1). Agreement among the reviewers was excellent, 95% (Cohen’s kappa 0.89; 95% CI 0.78 to 0.99). None of the studies had been able to blind patients nor therapists, but two studies [26, 38] had blinded the assessors. Three studies [26, 37, 40] did not use intention-to-treat analysis. In one study [25] groups differed at baseline and one study [40] did not publish measures of variability. On the Cochrane scale for clinical relevance [31] all studies were assigned scores of five out of five, except one [40], who lost one point due to the effect size being smaller than the MID for main outcomes. Complete PEDro scores and Cochrane relevance scores are available in Appendix 2 and 3.

**Table 1:** Characteristics and risk of bias of included studies

Author, year, country	Participants	Intervention (details in Table 2)	Control	Outcome measures	Follow-up period	PEDro score for quality (details in 2)	Cochrane score for clinical relevance (details in Appendix 3)
Bateman et al. 2014, United Kingdom [38]	*n* = 11, not responded to previous conservative treatment, on waiting list for operation, image verified rotator cuff tendinopathy or positive response to subacromial corticosteroid injection. Intervention 4 individuals, mean age 52 years; control_1 3 individuals, mean age 53 years; control_2 4 individuals, mean age 55 years.	Eccentric exercise; Intensity: pain reproduction	Resistance exercise (concentric phase only); Intensity matched experiment group	Pain: VAS; Function: Oxford Shoulder Score	8 weeks (post-treatment)	5/10	4/5
Blume et al. 2015, United States [35]	*n* = 34, shoulder pain, positive Neer’s, Hawkins or cross-body adduction test. Intervention 10 women 8 men, mean age 50.1 years (SD 16.9); control 10 women 6 men, mean age 48.6 years (SD 14.6).	Eccentric exercise; Intensity: 70–80% of 1RM	Resistance exercise (concentric phase only); Intensity matched experiment group	Pain: NPRS; Function: DASH	8 weeks (post-treatment)	8/10	5/5
Chaconas et al. 2017, United States [34]	*n* = 46, shoulder pain at least 3 months, 3 or more positive: Neer’s, Hawkins, empty can test, painful arc sign, shoulder external rotation pain, palpation pain supra/infraspinatus. Intervention 10 women 15 men, mean age 43.4 years (SD 17.9); control 9 women 12 men, mean age 48.4 years (SD 16.9).	Eccentric exercise; Intensity: 65% of 1RM	Resistance exercise; Intensity lower than experiment group	Pain: NPRS; Function: WORC	6 weeks (post-treatment) & 6 months (intermediate term follow-up)	6/10	5/5
Dejaco et al. 2017, Netherlands [37]	*n* = 36, subacromial pain at least 3 months, 2 of 3 positive: Neer’s, Hawkins, empty can test. Intervention 10 women 10 men, mean age 50.2 years (SD 10.8); control 7 women 9 men, mean age 48.6 years (SD 12.3).	Eccentric exercise; Intensity: pain reproduction	Resistance exercise; Intensity matched experiment group	Pain: VAS; Function: Constant-Murley Score	12 weeks (post-treatment) & 6 months (intermediate term follow-up)	7/10	5/5
Hallgren et al. 2014, Sweden [50]	*Reporting on the same study participant, intervention and control as Holmgren* et al *2012*			Pain: VAS; Function: Constant-Murley Score	1 year (long-term follow-up)	7/10	5/5
Holmgren et al. 2012, Sweden [26]	*n* = 97, diagnosed with primary SIS, shoulder pain at least 6 months, not responded to previous conservative treatment, on waiting list for operation, positive Neer’s injection test, 3 of 4 positive: Neer’s, Hawkins, empty can test, Patte’s manoeuvre. Intervention 14 women 37 men, mean age 52 years (SD 9); control 22 women 24 men, mean age 52 years (SD 8).	Eccentric exercise; Intensity: pain reproduction; Corticosteroid injection	Mobility exercise; Intensity lower than experiment group; Corticosteroid injection	Pain: VAS; Function: Constant-Murley Score	12 weeks (post-treatment)	7/10	5/5
Maenhout et al. 2013, Belgium [25]	*n* = 61, shoulder pain at least 3 months, painful arc sign, palpation pain supra/infraspinatus, 2 of 3 positive: Neer’s, Hawkins, empty can test and 2 of 4 painful resistance tests. Intervention 16 women 15 men, mean age 40.2 years (SD 12.9); control 20 women 10 men, mean age 39.4 years (SD 13.1).	Eccentric exercise; Intensity: pain reproduction	Resistance exercise; Intensity matched experiment group	Pain and function: SPADI	12 weeks (post-treatment)	6/10	5/5
Vallés-Carrascosa et al. 2018, Spain [36]	*n* = 22, diagnosed with SIS, positive painful arc sign. Intervention 8 women 3 men, mean age 57 years; control 4 women 7 men, mean age 60 years.	Eccentric exercise, pain up to 40–50 mm on VAS	Eccentric exercise, pain free execution	Pain: VAS; Function: Constant-Murley Score	4 weeks (post-treatment)	7/10	5/5

*SIS* Shoulder Impingement Syndrome, *VAS* Visual Analogue Scale, *NPRS* Numerical Pain Rating Scale, *DASH* Disabilities of the Arm, Shoulder and Hand questionnaire, *WORC* The Western Ontario Rotator Cuff index, *SPADI* Shoulder Pain and Disability Index

#### Participants

The seven included studies involved a total of 303 participants with subacromial impingement syndrome; 140 women and 156 men (one study [40] did not specify sex of the participants). Mean age of participants ranged from 39 to 60 years. Upon enrolment, pain duration varied from 3 months [25, 37, 39] to 6 months [26], with three studies [32, 38, 40] not specifying any timeframe. Pain intensity ranged from 12 mm VAS (“best pain”, rescaled from NPRS) to 70 mm VAS (“worst pain”). Disability levels at enrolment were mild to moderate. Detailed characteristics of included studies are presented in Table 1.

#### Interventions

Exercise regimens in the included studies showed both similarities and differences. All interventions focused on one or both of the following exercises: shoulder external rotation with shoulder in neutral position and shoulder abduction in the scapular plane with thumb pointing up (full can exercise). Elastic exercise bands and/or dumbbells were used in all studies. Duration of the intervention varied between four and 12 weeks and frequency varied between two times per week to two times per day. Exercise intensity was specified in two studies at 65% and 70–80% of 1RM (one repetition maximum) respectively [37, 38]. All other studies used pain reproduction to specify exercise intensity, i.e. allowing or encouraging pain during exercise that matched the pain normally felt by the participant in everyday activities, as long as it did not exceed 50 mm on VAS.

Comparators in all included studies consisted of other exercise interventions (one study [40] also included a second control group who did not receive any intervention, these participants are not included in the meta-analyses). In five studies [25, 37–40] the comparator was resistance exercise (concentric or concentric/eccentric). In one study [32] it was pain-free eccentric exercise (the experimental group performed painful eccentric exercise). In all aforementioned studies frequency, intensity and type of training were matched to the eccentric exercise protocol of the experimental group, except one study [37], in which the intensity was markedly lower. In the last study [26], the control group performed mobility exercises. In five of the studies [26, 32, 37–39], resistance training was complemented with stretching for both experimental and control group. In one of these [26], both experimental and control group also received a corticosteroid injection as well as advice on ergonomics and posture. Details on exercise regimens are presented in Table 2.

**Table 2:** Summary of eccentric training regimens

Study	Duration	Frequency	Intensity	Sets/reps	Equipment	Exercise(s)
Bateman et al. (2014) [38]	8 weeks	2 times/day	Same intensity for all participants, increased for all after 4 weeks, pain during execution allowed but not specified	3 × 15	Elastic exercise band: yellow and red	Full can with elastic band, only eccentric phase
Blume et al. (2015) [35]	8 weeks	2 times/week	Approx. 70–80% of 1RM, no increase of pain during execution	3 × 12	Dumbbells	Full can; sidelying ER; sidelying IR; supine protraction; sidelying horizontal abduction; sidelying abduction; prone shoulder extension; all with dumbbell, only eccentric phase + Pectoralis minor and posterior shoulder stretch, thoracic spine extension, pain-free AROM in flexion and abduction (accessory exercises done daily)
Chaconas et al. (2017) [34]	6 weeks	1 time/day	Approx. 65% of 1RM (15–18 RM), no increase of pain during execution	3 × 15	TheraBand: green, blue, black, silver, gold	ER with elastic band, only eccentric phase + Scapular retraction with elastic band, cross body posterior shoulder stretch
Dejaco et al. (2017) [37]	12 weeks	2 times/day	Pain reproduction, but not over 5 of 10 (NPRS) during execution	3 × 8–15	Duraband Servofit + dumbbell 1 kg (when needed)	ER with elastic band, only eccentric phase; empty can with/without dumbbell, only eccentric phase
Hallgren et al. (2014) [50]	*Same intervention as in Holmgren* et al *(2012), long-term results*					
Holmgren et al. (2012) [26]	12 weeks	2 times/day	Pain reproduction, but not over 5 of 10 (VAS/NPRS) during execution	3 × 15	Dumbbells + elastic exercise band	Full can with dumbbell, only eccentric phase; ER with elastic band, only eccentric phase + 3 scapular stabilization exercises; posterior shoulder stretch
Maenhout et al. (2013) [25]	12 weeks	2 times/day	Pain reproduction, but not over 5 of 10 (VAS/NPRS) during execution	3 × 15	Dumbbells + TheraBand: different colors	Full can with dumbbell, only eccentric phase + ER/IR with elastic band, no increase of pain during execution (3 × 10, once daily)
Vallés-Carrascosa et al. (2018) [32]	4 weeks (painful eccentric exercise)	5 times/week	Pain reproduction, but not over 40–50 mm VAS during execution	3 × 10	Dumbbells + elastic exercise band	Full can with dumbbell, only eccentric phase + ER/IR with elastic band; 2 scapular stabilization exercises; trapezius-stretch
	4 weeks (pain-free eccentric exercise)	5 times/week	No pain during execution, VAS = 0 mm	3 × 10	Dumbbells + elastic exercise band	Full can with dumbbell, only eccentric phase + ER/IR with elastic band; 2 scapular stabilization exercises; trapezius-stretch

Full can/empty can = shoulder abduction in scapular plane (scaption) with a thumbs up/thumbs down position; ER/IR external/internal rotation with neutral shoulder and elbow flexed 90°, *RM* Repetition Maximum, *VAS* Visual Analogue Scale, *NPRS* Numerical Pain Rating Scale

#### Outcome measures

Pain and function were measured in all included studies. In four studies, as well as the one-year follow up [26, 32, 39–41], pain was measured using a 100 mm VAS [34]. In two studies [37, 38], a 10-point NPRS [42] was used. In one study [25] pain was measured with the Shoulder Pain and Disability Index (SPADI [43]) pain subscale.

For function, five different instruments were used in the seven studies: the Oxford Shoulder Score (OSS [44]), the Disability of the Arm, Shoulder and Hand (DASH [43]), the Western Ontario Rotator Cuff (WORC) index [45], the Constant-Murley (CM) Score [46], and the SPADI total score [43]. All measures have been validated.

#### Summary of findings

Summary of findings for the main comparisons are presented in Table 3 and described below for each outcome.

**Table 3:** Summary of findings for the comparison eccentric exercise versus control exercise for subacromial impingement syndrome

Outcomes, time frame	Absolute effect estimates (95% CI)		№ of participants (studies)	Certainty in effect estimates (GRADE)	Conclusion
	Control exercise	Eccentric exercise			
Pain: post-treatment (6–12 weeks) Measured by VAS or NPRS, converted to VAS 0–100 mm (lower better) MID: 15 mm	Mean post-treatment pain ranged across control groups from 15.0 to 63.9 mm	Mean post-treatment pain in the experimental group was **12.3 mm lower** (17.8 lower to 6.8 lower)	281 (6 studies)	Low^a, b^	Eccentric exercise may provide a small but likely not important reduction in pain post-treatment compared with other types of exercise.
Pain: intermediate to long-term (6–12 months) Measured by VAS or NPRS, converted to VAS 0–100 mm (lower better) MID: 15 mm	Mean intermediate/long-term pain ranged across control groups from 18.0 to 52.1 mm	Mean intermediate/long-term pain in the experimental group was **4.9 mm lower** (15.4 lower to 5.6 higher)	167 (3 studies)	Low^a, b^	Eccentric exercise may result in little or no important difference in pain compared with other types of exercise.
Function: post-treatment (6–12 weeks) Multiple scales of various range	N/A	Standardised mean post-treatment function in the experimental group was **0.10 SMD units better** (0.79 better to 0.58 worse)	281 (6 studies)	Very low^a, b, c^	It is uncertain whether eccentric exercise improves function more than other types of exercise post-treatment follow-up.
Function: intermediate to long-term (6–12 months) Multiple scales of various range	N/A	Standardised mean intermediate/long-term function in the experimental group was **0.28 SMD units worse** (0.67 better to 1.24 worse)	167 (3 studies)	Very low^a, b, c^	It is uncertain whether eccentric exercise improves function more than other types of exercise at intermediate/long-term follow-up.

GRADE Working Group grades of evidence

High certainty: We are very confident that the true effect lies close to that of the estimate of the effect

Moderate certainty: We are moderately confident in the effect estimate: The true effect is likely to be close to the estimate of the effect, but there is a possibility that it is substantially different

Low certainty: Our confidence in the effect estimate is limited: The true effect may be substantially different from the estimate of the effect

Very low certainty: We have very little confidence in the effect estimate: The true effect is likely to be substantially different from the estimate of effect

GRADE Grading of Recommendations Assessment, Development and Evaluation, *MID* minimal important difference, *VAS* Visual Analogue Scale, *NPRS* Numerical Pain Rating Scale, *N/A* not applicable

^a^Downgraded one level due to serious risk of bias (mainly due to lack of blinding)

^b^Downgraded one level due to serious imprecision (high heterogeneity in magnitude and direction of effect across studies, wide CIs, small study sizes)

^c^Downgraded one level due to clear inconsistency of results

### Effects of eccentric exercise on pain

**Fig. 2:**
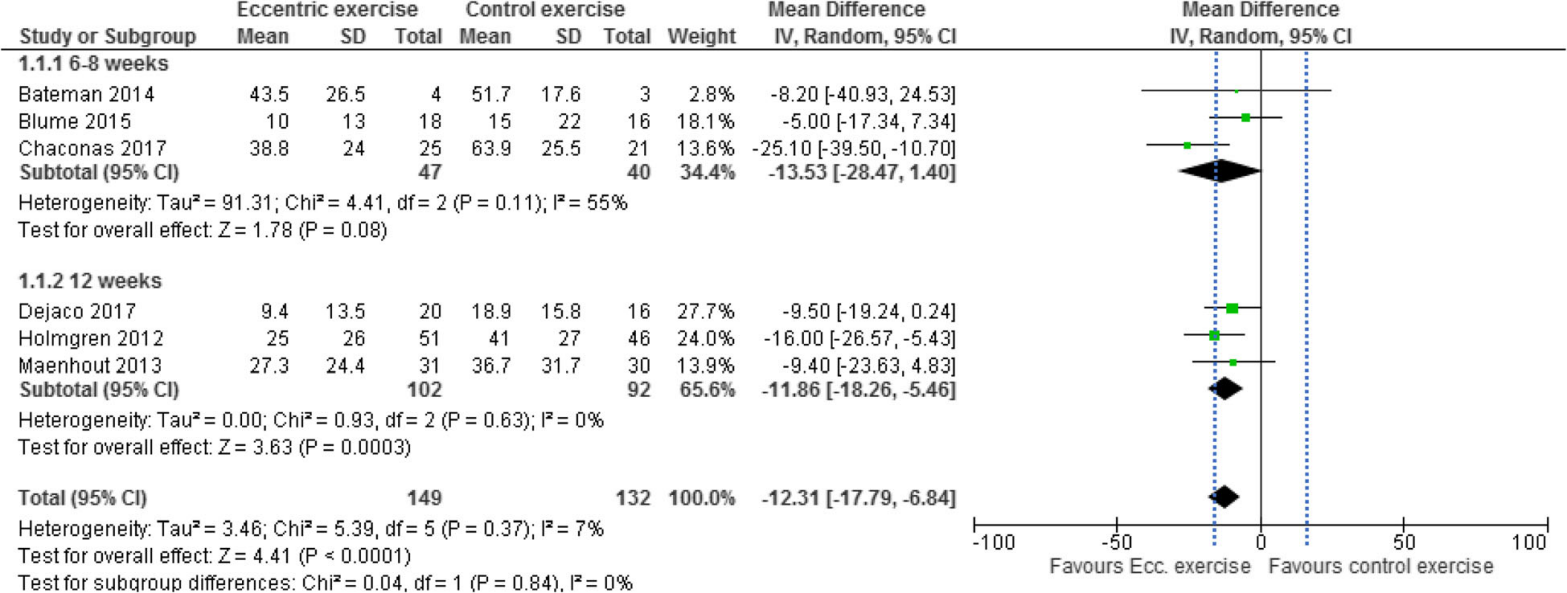
Forest plot of post-treatment effects on pain, sub-grouped by treatment length of six to eight weeks and treatment length of 12 weeks. Dashed vertical line denotes minimal important difference (VAS 15 mm). IV = inverse-variance, VAS = visual analogue scale

Effects on all measures of pain are presented in Appendix 4. Although treatment lengths in the studies varied between four and 12 weeks, six of the included studies [25, 26, 37–40] measured post-treatment pain and were considered to be of sufficient clinical homogeneity to be combined. Intermediate to long-term effects (6–12 months follow-up) were measured in three studies [37, 39, 41].Six studies with 281 participants were included in the meta-analysis of post-treatment effects on pain. In the three studies [37, 38, 40] with treatment lengths of six to 8 weeks, eccentric exercise provided no significant reduction in pain compared with other exercise regimens: MD -13.5 (95% CI − 28.5 to 1.4, I^2^ = 55%, *p* = 0.08). In the three studies [25, 26, 39] with treatment length of 12 weeks, eccentric exercise provided significant reduction in pain compared with other exercise regimens: MD -11.9 (95% CI − 18.2 to − 5.5, I^2^ = 0%, *p* < 0.001). In total, post-treatment pain was significantly lower after eccentric exercise compared with other exercise regimens: combined MD -12.3 (95% CI − 17.8 to − 6.8, I^2^ = 7%, *p* < 0.001) (Fig. 2). However, because the MD did not surpass the MID of 15 mm on VAS [34], the effect was not considered clinically relevant.

**Fig. 3:**
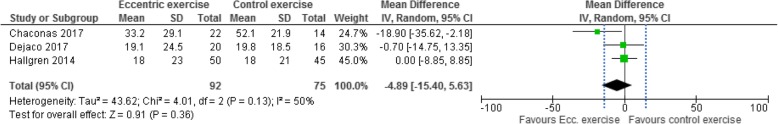
Forest plot of intermediate to long-term (6–12 months) effects on pain. Dashed vertical lines denote minimal important difference (VAS 15 mm). IV = inverse-variance, VAS = visual analogue scale

Three studies [37, 39, 41] with 167 participants were included in the meta-analysis of intermediate to long-term effects on pain, assessed at six or 12 months. Eccentric exercise did not provide an overall significant intermediate to long-term reduction in pain compared with other exercise regimens: MD -4.9 (95% CI − 15.4 to 5.6, I^2^ = 50%, *p* = 0.36) (Fig. 3).

Certainty of evidence was assessed as low, for both short-term and intermediate to long-term effects on pain. We downgraded one level for risk of bias because all studies are at unclear risk of bias, mainly due to lack of blinding. We downgraded one level for imprecision because the 95% CI included both no clinically relevant effect (MID less than 15 mm on VAS) and clinically relevant effect (MID greater than 15 mm on VAS).

The study that compared painful versus pain-free eccentric exercise [32] only measured effects immediately after the four-week long intervention, and showed no significant difference in pain between experimental and control group. According to the PEDro scale it was assessed as having a low risk of bias.

No studies reported any negative effects of (increased) pain during exercise, nor any other side effects.

### Effects of eccentric exercise on function

**Fig. 4:**
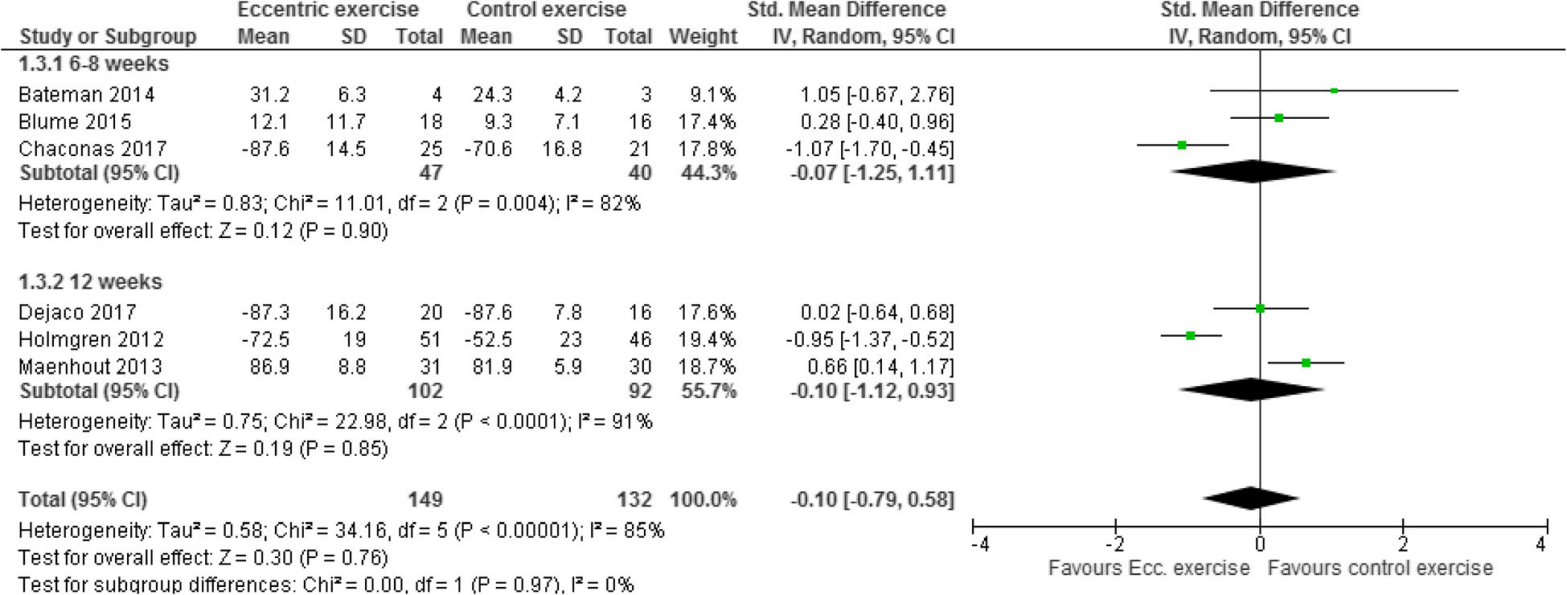
Forest plot of post-treatment effects on function, sub-grouped by treatment length of six to eight weeks and treatment length of 12 weeks. Because the scales used in Dejaco 2017 and Holmgren 2012 go in the opposite direction (i.e. higher is better) than the ones used in the other studies, the mean values were multiplied by − 1. As a rule of thumb, a standardised mean difference of 0.2 represents a small difference, 0.5 a moderate, and 0.8 a large difference. IV = inverse-variance

Effects on function are presented in Appendix 5. Six studies with 281 participants were included in the meta-analysis of post-treatment effects on function; three studies [37, 38, 40] with treatment lengths of six to 8 weeks and three studies [25, 26, 39] with treatment length of 12 weeks. The meta-analysis shows considerable variation in effects. Eccentric exercise provided no significant post-treatment improvement in function compared with other exercise regimens, regardless of whether the intervention lasted six to 8 weeks or 12 weeks: SMD -0.10 (95% CI − 0.79 to 0.58, I^2^ = 85%, *p* = 0.76) (Fig. 4).

**Fig. 5:**
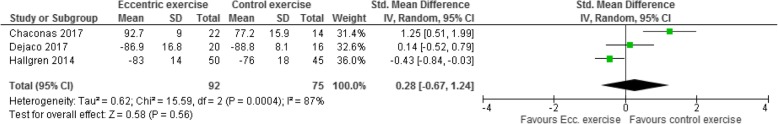
Forest plot of intermediate to long-term effects on function. Because the scales used in Dejaco 2017 and Hallgren 2014 go in the opposite direction (i.e. higher is better) than the one used in the other study, the mean values were multiplied by − 1. As a rule of thumb, a standardised mean difference of 0.2 represents a small difference, 0.5 a moderate, and 0.8 a large difference. IV = inverse-variance

Three studies [37, 39, 41] with 167 participants were included in the meta-analysis of intermediate to long-term effects on function, assessed at six or 12 months. The meta-analysis showed considerable variation in effects. Eccentric exercise provided no significant intermediate or long-term improvement in function compared with other exercise regimens: SMD 0.28 (95% CI − 0.67 to 1.24, I^2^ = 87%, *p* = 0.56) (Fig. 5).

Certainty of evidence for short-term and intermediate to long-term effects on function was assessed as very low. We downgraded one level for risk of bias because all studies are at unclear risk of bias, mainly due to lack of blinding. We downgraded one level for imprecision because the 95% CI favoured both eccentric and control exercise. We also downgraded one level for inconsistency because of the variance across studies in both direction and size of the effect.

## Discussion

Based on six trials of low to moderate risk of bias, including a total of 281 patients with subacromial impingement syndrome and comparing eccentric exercise to other types of exercise, the findings of this review and meta-analysis suggest that eccentric exercise provides slightly better effect on pain but not on function compared with other exercise. However, while the meta-analysis for pain showed that eccentric exercise reduced pain significantly more than other exercise post-treatment, this difference was not greater than the MID, and the difference was not sustained at intermediate to long-term follow-ups. The meta-analysis for function showed no difference between eccentric exercise and other exercise. The certainty of evidence in these findings is low to very low, due to study limitations, imprecision and, for function, inconsistency across the studies. Based on one trial of low risk of bias, including 22 participants, painful eccentric exercise did not yield any significant difference compared with pain-free eccentric exercise.

Of the individual studies, Holmgren et al. [26] showed that eccentric exercise was better than non-loading mobility exercises for both pain and function and Chaconas et al. [37] showed a statistically significant and clinically important between-group difference in pain and function between strength training groups, favouring eccentric exercise. The remaining four RCTs [25, 38–40] did not find any such significant difference between groups, although a trend can be discerned across the studies in favour of eccentric exercise.

Our findings lend further support to earlier reviews that have investigated various types of exercise for subacromial impingement syndrome [14, 22–24]. In their review of eccentric exercise for subacromial impingement and lateral epicondylalgia, Ortega-Castillo & Medina-Porqueres [22] concluded that eccentric exercise may reduce pain and improve strength in upper limb tendinopathies, but that it was questionable whether it was more effective than other forms of treatment. Valier et al. [23] surmised in their “critically appraised topic” that there were conflicting results whether the addition of an eccentric exercise component in shoulder rehabilitation programs would reduce pain or increase function. Both those reviews only included two RCTs [25, 26] of the ones we included in our review. Hanratty et al. [14] who included both concentric and eccentric exercise interventions in their review concluded that “there was strong evidence that exercise decreases pain and improves function at short-term follow-up”. However, because it included different types of exercise it is not completely comparable to our review.

The aggregated difference in pain reduction was similar for the six to 8 week interventions and those of 12 weeks, which may indicate that the shorter treatment length is sufficient to address pain. The fact that significance was reached for the longer intervention period but not for the shorter is likely due to the low power of those trials. None of the subgroups showed improvement greater than the minimally important difference (15 mm VAS [34]). In the 12-week study by Maenhout et al. [25], the greatest improvement was reported after the first 6 weeks, further supporting that interventions of six to 8 weeks could be sufficient to obtain measurable effects.

Although most studies focused on short-term effects of eccentric exercise, two of them reported a six-month follow-up [37, 39] and one was a one-year follow-up [41]. All found that the MD in pain between eccentric exercise and control was smaller at the follow-up, and the non-difference in function remained. Although it is likely that both experimental and control groups improved spontaneously over time, the findings suggest that after 1 year it does not matter which type of exercise you perform.

Chaconas et al. [37] have suggested that the purpose of eccentric exercise is to use training intensities that are so high that the exercises cannot be performed in the concentric phase. This is possible because individuals are on average 20% stronger during eccentric muscle contractions than during concentric contractions [47, 48]. It also has been reported that the experience of pain is less, and the reversion of pain faster, during eccentric exercises than during concentric ones [49]. Previous research also has shown that maximum intensity eccentric knee extensions (but not maximum intensity concentric extensions) may increase satellite cell activation necessary for muscle repair [50], and eccentric bench-press at 90% of 1RM may double growth hormone release, also associated with muscle repair and hypertrophy, compared with both concentric and eccentric bench-presses at 70% of 1RM [51]. However, in none of the included studies in this review were the eccentric training loads so high that they could not also be performed concentrically. In the study by Blume et al. [38], concentric exercises were even used to calculate the intensity of the eccentric ones, without adjusting for difference in strength. Only two of the seven included studies clearly defined training intensities at all; Blume et al. [38] used an intensity of 70–80% of 1RM for both experimental and control group, and Chaconas et al. [37] used 65% of 1RM for the eccentric exercise group. The remaining study protocols used pain reproduction to define the training intensity. Intensity of the control group exercises were relatively well matched to that of the experiment group, with two exceptions [26, 37]; these were also the two studies that showed the biggest between-group differences. It might therefore be speculated, that what matters most is not the type of exercise performed, but at what intensity.

As of yet, no studies have been published in which the effects of heavy (> 85% of 1RM) eccentric training of the rotator cuff have been investigated, but one study looking at heavy resistance training (concentric/eccentric) in patients with rotator cuff tendinopathies did not find any difference between higher (85% of 1RM) and lower (50% of 1RM) intensity resistance training [52]. Future research that aims to compare eccentric and other resistance exercise in patients with subacromial impingement syndrome should therefore focus on, and clearly define, heavy (80–90% of 1RM) eccentric exercise. Intensity should be individualised and defined as percentage of 1RM rather than aiming for pain reproduction or pain up to a certain level, since this review shows that a certain experience of pain does not indicate a better outcome than other levels of pain, or no pain at all [25, 32].

Of the seven included studies, aiming to investigate eccentric exercise for tendinopathy, only three [25, 37, 40] actually controlled whether the shoulder pain originated from a muscle tendon. This included pain on palpation of the supra- or infraspinatus tendon as a possible or required inclusion criterion, or ultrasound or MRI (magnetic resonance imaging) verified rotator cuff tendinopathy. It is thus not possible to know the proportion of participants in the different studies in whom the pain originated from a muscle tendon, and the proportion in whom structural changes to the acromion process or bursitis were the primary pathogeneses. Future studies in this field therefore need to specifically control for and verify tendinopathies in order to investigate whether there is a correlation between pain from one or more muscle tendons of the rotator cuff (or the long head of the biceps brachii) and the effects of eccentric exercise, compared with patients without a clear tendon involvement. It is likely that optimal exercise strategy will vary depending on affected tendon or other primary pathogenesis.

This review is based on small to medium-sized trials, of which the smallest only had 11 participants. A major limitation in all studies is the lack of blinding of both participants and therapists, a common problem in physiotherapy trials. Most studies also failed to blind the outcome assessors. Other limitations are the large variation in the exercise protocols and generally poor definition of training intensity, leading to high heterogeneity and precluding any conclusions to be drawn about the most effective exercise regimen. For example, it is possible that the results in the studies by Chaconas et al. [37] and Holmgren et al. [26] came from the greater training intensities rather than the specific type of exercise. It could therefore be argued that these studies should have been excluded from the meta-analyses. We instead chose to include them, partly because the aim of this review was to compare eccentric exercise to any intervention, regardless of what type, but also because high intensity loading can be seen as the hallmark of eccentric training and not a confounding factor. The high heterogeneity among the trials also became apparent when we pooled the outcome data for function. Besides the clinical heterogeneity in exercise protocols, there was also a wide range of outcome measures used to evaluate function, further contributing to the high heterogeneity. It could be questioned whether pooling the data for function was appropriate, but we determined that it was useful both for illustrating the heterogeneity and for assessing whether there was sufficient inconsistency among studies to downgrade the certainty of evidence.

A potential limitation with our review is that only three databases were searched, and no grey literature was searched. However, we chose the most relevant databases and had no reason to believe that any studies not published and indexed in either of the searched databases would exist; hence, we limited our search strategy to those databases. We did not apply a minimal important difference on the effects on function because we used SMD in our calculations to the heterogeneity in outcome measures. However, the difference between groups was not significant at any of the time points. Strengths of the review are the rigorous methodology, that we appraised evidence from randomised controlled trials only, that we performed meta-analyses, and that we assessed the body of evidence using GRADE.

In conclusion, evidence of low certainty suggests that eccentric exercise may provide a small but likely not clinically important reduction in pain post-treatment compared with other types of exercise in patients with subacromial impingement syndrome. At intermediate to long-term follow-up, eccentric exercise may result in little or no important difference in pain compared with other types of exercise. It is uncertain whether eccentric exercise improves function more than other types of exercise post-treatment and at intermediate to long-term follow-up in patients with subacromial impingement syndrome (very low certainty of evidence). Pain during exercise does not seem to provide greater improvement in pain or function compared with pain-free exercise. On the other hand, no negative effects have been observed. Eccentric training regimens have shown both similarities and diversity. Intervention durations of six to 8 weeks have been shown to be similar in effectiveness as an intervention duration of 12 weeks. For frequency and intensity no conclusions can be made, but it seems that exercise at higher intensities might yield better results. When it comes to exercise type, only shoulder external rotation with neutral shoulder and the full can exercise have been studied. It is likely that the optimal exercise will vary depending on underlying tendinopathy. Further research as outlined above is necessary.

## Data Availability

The datasets used and/or analysed during the current study are available from the original source articles or from the corresponding author on reasonable request.

## Appendix 1

### Search strategies and search results

**Database:** PubMed

**Date:** 6 March 2019.

**No. of results:** 37

**Table:**

Search	Query	Items found
#6	Search (((((“shoulder impingement” OR “subacromial impingement” OR “subacromial pain” OR “rotator cuff”[tiab])) OR subacromial impingement syndrome[mh]) OR rotator cuff[mh])) AND (“eccentric exercise” OR “eccentric exercises” OR “eccentric training” OR “eccentric strengthening” OR “eccentric strength training”[tiab])	37
#5	Search “eccentric exercise” OR “eccentric exercises” OR “eccentric training” OR “eccentric strengthening” OR “eccentric strength training”[tiab]	1981
#4	Search (((“shoulder impingement” OR “subacromial impingement” OR “subacromial pain” OR “rotator cuff”[tiab])) OR subacromial impingement syndrome[mh]) OR rotator cuff[mh]	12594
#3	Search rotator cuff[mh]	5788
#2	Search subacromial impingement syndrome[mh]	1654
#1	Search “shoulder impingement” OR “subacromial impingement” OR “subacromial pain” OR “rotator cuff”[tiab]	11637

**Database:** The Cochrane Library.

**Date:** 6 March 2019.

**No. of results:** 27

Cochrane Reviews [2].

Cochrane Protocols (0).

Trials [25].

Editorials (0).

Special Collections (0).

Clinical Answers (0).

Other Reviews (0).

**Table:**

**ID**	**Search**	**Hits**
#1	(“shoulder impingement” OR “subacromial impingement” OR “subacromial pain” OR “rotator cuff”):ti,ab,kw (Word variations have been searched)	1425
#2	MeSH descriptor: [Subacromial impingement syndrome] explode all trees	280
#3	MeSH descriptor: [Rotator Cuff] explode all trees	318
#4	#1 OR #2 OR #3	1425
#5	(“eccentric exercise” OR “eccentric exercises” OR “eccentric training” OR “eccentric strengthening” OR “eccentric strength training”):ti,ab,kw (Word variations have been searched)	694
#6	#4 AND #5	27

**Database:** PEDro.

**Date:** 6 March 2019.

**No. of results:** 4.

**Table:**

**Search**	**Records found**
Abstract and title: (shoulder impingement” OR “subacromial impingement” OR “subacromial pain” OR “rotator cuff) AND eccentric, Clinical trial	4

**Reference lists**

A comprehensive search of reference lists brought **1** new records.

## Appendix 2

**Table 4:** PEDro scores for the included studies

Study	Eligibility criteria and source	Random allocation	Concealed allocation	Groups similar at baseline	Participant blinding	Therapist blinding	Assessor blinding	< 15% dropouts	Intention- to-treat analysis	Between-group difference reported	Point estimates and variability reported	Total score (0 to 10)
Bateman et al. (2014) [38]	Yes	Yes	Yes	Yes	No	No	No	Yes	No	Yes	No	5
Blume et al. (2015) [35]	Yes	Yes	Yes	Yes	No	No	Yes	Yes	Yes	Yes	Yes	8
Chaconas et al. (2017) [34]	Yes	Yes	No	Yes	No	No	Yes	Yes	No	Yes	Yes	6
Dejaco et al. (2017) [37]	Yes	Yes	Yes	Yes	No	No	No	Yes	Yes	Yes	Yes	7
Hallgren et al. (2014) [50]	Yes	Yes	Yes	Yes	No	No	Yes	Yes	No	Yes	Yes	7
Holmgren et al. (2012) [26]	Yes	Yes	Yes	Yes	No	No	Yes	Yes	No	Yes	Yes	7
Maenhout et al. (2013) [25]	Yes	Yes	Yes	No	No	No	No	Yes	Yes	Yes	Yes	6
Vallés-Carrascosa et al. (2018) [32]	Yes	Yes	Yes	Yes	No	No	No	Yes	Yes	Yes	Yes	7

## Appendix 3

**Table 5:** Cochrane scale for clinical relevance

Study	Are the patients described in detail so that you can decide whether they are comparable to those that you see in your practice?	Are the interventions and treatment settings described well enough so that you can provide the same for your patients?	Were all clinically relevant outcomes measured and reported?	Is the size of the effect clinically important?	Are the likely treatment benefits worth the potential harms?	Total score (0 to 5)
Bateman et al. (2014) [38]	Yes	Yes	Yes	No	Yes	4
Blume et al. (2015) [35]	Yes	Yes	Yes	Yes	Yes	5
Chaconas et al. (2017) [34]	Yes	Yes	Yes	Yes	Yes	5
Dejaco et al. (2017) [37]	Yes	Yes	Yes	Yes	Yes	5
Hallgren et al. (2014) [50]	Yes	Yes	Yes	Yes	Yes	5
Holmgren et al. (2012) [26]	Yes	Yes	Yes	Yes	Yes	5
Maenhout et al. (2013) [25]	Yes	Yes	Yes	Yes	Yes	5
Vallés-Carrascosa et al. (2018) [32]	Yes	Yes	Yes	Yes	Yes	5

## Appendix 4

**Table 6:** Outcome table for pain

Study	Number of participants	Withdrawals/dropouts	Intervention Eccentric exercise (painful eccentric exercise)	Control Other exercise types (non-painful eccentric exercise)	Comments
Bateman et al. (2014) [38]	I: 4 C1: 3 (concentric group) C2:4 (no exercise)	I: 1 C1: 0 C2: 1	VAS Baseline: 53.0 (SD 14.2) Post-treatment (8 weeks): 43.5 (SD 26.5) Δ −9.5	VAS Baseline: 42.3 (SD 6.9) Post-treatment (8 weeks): 51.7 (SD 17.6) Δ 9.4	Between-group difference reported for intervention and concentric exercise group (C1). Second control group (C2) not reported because no intervention was given. Visual analog scale, 0–100 mm, where 100 indicates worst possible pain.
			*Between-group difference in change:* *Δ 18.9; in favour of eccentric exercise (ns)*		
Blume et al. (2015) [35]	I: 18 C: 20	I: 0 C: 4	NPRS Baseline: 2.0 (SD 1.8) Post-treatment (8 weeks): 1.0 (SD 1.3) Δ −1.0	NPRS Baseline: 2.1 (SD 1.6) Post-treatment (8 weeks): 1.5 (SD 2.2) Δ −0.6	Numerical Pain Rating Scale. Range 0–10, where 10 indicates worst pain. Data for 8 weeks were provided via e-mail.
			*Between-group difference in change:* *Δ 0.4; in favour of eccentric exercise (ns)*		
Chaconas et al. (2017) [34]	I: 25 C: 21	I: 0 C: 2	NPRS Best Pain: Baseline: 1.64 (SD 1.71) Post-treatment (6 weeks): 1.00 (SD 1.47) Δ −0.64 At 6 months: 0.54 (SD 1.18) Δ −1.10 Average Pain: Baseline: 3.72 (SD 2.03) Post-treatment (6 weeks): 1.40 (SD 1.68) Δ −2.32 At 6 months: 1.04 (SD 1.62) Δ −2.68 Worst pain: Baseline: 7.00 (SD 1.78) Post-treatment (6 weeks): 3.88 (SD 2.40) Δ −3.12 At 6 months: 3.32 (SD 2.91) Δ −3.68	NPRS Best Pain: Baseline: 1.22 (SD 1.28) Post-treatment (6 weeks): 1.13 (SD 1.25) Δ − 0.09 At 6 months: 1.00 (SD 1.30) Δ − 0.22 Average Pain: Baseline: 3.30 (SD 1.61) Post-treatment (6 weeks): 2.70 (SD 2.03) Δ − 0.60 At 6 months: 2.14 (SD 1.83) Δ − 1.16 Worst pain: Baseline: 7.00 (SD 2.09) Post-treatment (6 weeks): 6.39 (SD 2.55) Δ − 0.61 At 6 months: 5.21 (SD 2.19) Δ − 1.79	
			*Between-group difference in change at 6 weeks (best pain):* *Δ 0.55; in favour of eccentric exercise (ns)* *Between-group difference in change at 6 months (best pain):* *Δ 0.88; in favour of eccentric exercise (p = 0.02)* *Between-group difference in change at 6 weeks (average pain):* *Δ 1.72; in favour of eccentric exercise (p < 0.001)* *Between-group difference in change at 6 months (average pain):* *Δ 1.52; in favour of eccentric exercise (p = 0.02)* *Between-group difference in change at 6 weeks (worst pain):* *Δ 2.51; in favour of eccentric exercise (p < 0.001)* *Between-group difference in change at 6 months (worst pain):* *Δ 1.89; in favour of eccentric exercise (p = 0.006)*		
Dejaco et al. (2017) [37]	I: 20 C: 16	I: 1 C: 1	VAS Baseline: 39.0 (SD 18.5) Post-treatment (12 weeks): 9.4 (SD 13.5) Δ − 29.6 At 6 months: 19.1 (SD 24.5) Δ − 19.9	VAS Baseline: 42.0 (SD 27.0) Post-treatment (12 weeks): 18.9 (SD 15.8) Δ − 23.1 At 6 months: 19.8 (SD 18.5) Δ − 22.3	
			*Between-group difference in change at 12 weeks:* *Δ 6.5; in favour of eccentric exercise (ns)* *Between-group difference in change at 6 months:* *Δ 2.4; in favour of control (ns)*		
Hallgren et al. (2014) [50]	I: 51 C: 46	I: 1 C:1	VAS Pain at rest: Baseline: 15 (SD 19) At 1 year: 2 (SD 6) Δ − 13 Pain during activity: Baseline: 61 (SD 22) At 1 year: 18 (SD 23) Δ −43 Pain at night: Baseline: 46 (SD 28) At 1 year: 12 (SD 19) Δ − 34	VAS Pain at rest: Baseline: 20 (SD 21) At 1 year: 4 (SD 13) Δ − 16 Pain during activity: Baseline: 66 (SD 20) At 1 year: 18 (SD 21) Δ −48 Pain at night: Baseline: 40 (SD 30) At 1 year: 14 (SD 21) Δ − 26	Long-term follow-up of the study by Holmgren et al. 2012. NB: 12 patients in the intervention group and 29 in the control group underwent surgery but were still included in 1-year follow-up.
			*Between-group difference in change (pain at rest):* *Δ 3; in favour of control (ns)* *Between-group difference in change (pain during activity):* *Δ 5; in favour of control (ns)* *Between-group difference in change (pain at night):* *Δ 8; in favour of eccentric exercise (ns)*		
Holmgren et al. (2012) [26]	I: 51 C: 46	I: 0 C: 0	VAS Pain at rest: Baseline: 15 (SD 19) Post-treatment (12 weeks): 10 (SD 14) Δ − 5 Pain during activity: Baseline: 61 (SD 22) Post-treatment (12 weeks): 25 (SD 26) Δ − 36 Pain at night: Baseline: 46 (SD 28) Post-treatment (12 weeks): 15 (SD 22) Δ − 31	VAS Pain at rest: Baseline: 20 (SD 21) Post-treatment (12 weeks): 20 (SD 25) Δ 0 * Pain during activity: Baseline: 66 (SD 20) Post-treatment (12 weeks): 41 (SD 27) Δ − 25 Pain at night: Baseline: 40 (SD 30) Post-treatment (12 weeks): 27 (SD 27) Δ − 13	* Holmgren reports an erroneous value for within group change for VAS at rest for the control group (− 5).
			*Between-group difference in change (pain at rest):* *Δ 5; in favour of eccentric exercise (ns)* *Between-group difference in change (pain during activity):* *Δ 11; in favour of eccentric exercise (ns)* *Between-group difference in change (pain at night):* *Δ 18; in favour of eccentric exercise (95% CI − 30.9 to − 7.2)*		
Maenhout et al. (2013) [25]	I: 31 C: 30	I: 4 C: 5	SPADI Pain subscale Baseline: 25.8 (SD 10.4) Post-treatment (12 weeks): 11.0 (SD 9.5) Δ − 14.8	SPADI Pain subscale Baseline: 28.4 (SD 12.4) Post-treatment (12 weeks): 11.0 (SD 11.5) Δ − 17.4	Pain was measured using the SPADI. Data were provided via e-mail so that we could extract data for the pain subscale. Scale ranges from 0 to 50, higher score indicates worse pain.
			*Between-group difference in change:* *Δ 2.6; in favour of control (ns)*		
Vallés-Carrascosa et al. (2018) [32]	I: 11 C: 11	I: 0 C: 0	VAS Baseline: 37.0 (32.0; 79.0) Post-treatment (4 weeks): 12.0 (3.0; 30.0) Δ − 25.0	VAS Baseline: 55.0 (48.0; 68.0) Post-treatment (4 weeks): 28.0 (18.0; 37.0) Δ − 27.0	Median (1st; 3rd quartile)
			*Between-group difference in change:* *Δ 2; in favour of non-painful eccentric exercise (ns)*		

*RCT* randomized controlled trial, *VAS* Visual Analogue Scale, *NPRS* Numerical Pain Rating Scale, *SPADI* Shoulder Pain and Disability Index, *ns* not significant, *CI* confidence interval

## Appendix 5

**Table 7:** Outcome table for function

Study	Number of participants	Withdrawals/dropouts	Intervention Eccentric exercise (painful eccentric exercise)	Control Other exercise types (non-painful eccentric exercise)	Comments
Bateman et al. (2014) [38]	I: 4 C1: 4 (concentric group) C2:3 (no exercise)	I: 1 C1: 1 C2: 0	Oxford Shoulder Score Baseline: 34.0 (SD 5.3) Post-treatment (8 weeks): 31.3 (SD 6.3) Δ − 2.7	Oxford Shoulder Score Baseline: 24.0 (SD 3.7) Post-treatment (8 weeks): 24.3 (SD 4.2) Δ + 0.3	Control group 2 (C2) excluded because no treatment was given. Function measured with the 12-item Oxford Shoulder Score. Range 12–60, where 12 indicates no pain and normal function and higher is worse. MID 6 points [43].
			*Between-group difference in change:* *Δ 3.0; in favor of eccentric exercise (ns)*		
Blume et al. (2015) [35]	I: 18 C: 20	I: 0 C: 4	DASH Baseline: 25.0 (SD 10.6) Post-treatment (8 weeks): 12.1 (SD 11.7) Δ − 12.9	DASH Baseline: 21.2 (SD 6.5) Post-treatment (8 weeks): 9.3 (SD 7.1) Δ − 11.9	Function measured with the 30-item DASH questionnaire. Range 0–150, higher is worse. MID = 10.5 points [40].
			*Between-group difference in change:* *Δ 1.0; in favor of eccentric exercise (ns)*		
Chaconas et al. (2017) [34]	I: 25 C: 21	I: 0 C: 2	WORC Baseline: 66.6 (SD 16.0) Post-treatment (6 weeks): 87.6 (SD 14.5) Δ + 21.0 At 6 months: 92.7 (SD 9.0) Δ + 26.1	WORC Baseline: 65.4 (SD 14.1) Post-treatment (6 weeks): 70.6 (SD 16.8) Δ + 5.2 At 6 months: 77.2 (SD 15.9) Δ + 11.8	Function measured with WORC. Total range 0–2100 (aggregate score of 6 total sub-domains), here reported as scores out of 100 i.e. a percentage of normal score, where higher is better. MID = 275 points [39].
			*Between-group difference in change at 6 weeks:* *Δ 17.0; in favor of eccentric exercise (p < 0.001)* *Between-group difference in change at 6 months:* *Δ 15.1; in favor of eccentric exercise (p = 0.007)*		
Dejaco et al. (2017) [37]	I: 20 C: 16	I: 1 C: 1	Constant-Murley Score Baseline: 72.5 (SD 17.7) Post-treatment (12 weeks): 87.3 (SD 16.2) Δ + 14.8 At 6 months: 86.9 (SD 16.8) Δ + 14.4	Constant-Murley Score Baseline: 78.9 (SD 8.5) Post-treatment (12 weeks): 87.6 (SD 7.8) Δ + 8.7 At 6 months: 88.8 (SD 8.1) Δ + 9.9	Function measured with Constant-Murley Score. Four subscales (pain, ADL, ROM, strength). Total range 0–100, where 100 indicates excellent shoulder function. MID = 17 points [41].
			*Between-group difference in change at 12 weeks:* *Δ 6.1; in favor of eccentric exercise (ns)* *Between-group difference in change at 6 months:* *Δ 4.5; in favor of eccentric exercise (ns)*		
Hallgren et al. (2014) [50]	I: 51 C: 46	I: 1 C:1	Constant-Murley Score Baseline: 48.5 (SD 15.0) 1-year follow up: 83.0 (SD 14.0) Δ + 34.5	Constant-Murley Score Baseline: 43.5 (SD 15.0) 1-year follow up: 76.0 (SD 18.0) Δ + 32.5	Long-term follow-up of the study by Holmgren et al. 2012. Baseline values from the Holmgren et al. paper.
			*Between-group difference in change:* *Δ 2; in favor of eccentric exercise (ns)*		
Holmgren et al. (2012) [26]	I: 51 C: 46	I: 0 C: 0	Constant-Murley Score Baseline: 48.5 (SD 15.0) Post-treatment (12 weeks): 72.5 (SD 19.0) Δ + 24	Constant-Murley Score Baseline: 43.5 (SD 15.0) Post-treatment (12 weeks): 52.5 (SD 23.0) Δ + 9	
			*Between-group difference in change:* *Δ 15; in favor of eccentric exercise (95% CI 8.5 to 20.6)*		
Maenhout et al. (2013) [25]	I: 31 C: 30	I: 4 C: 5	SPADI Baseline: 42.0 (SD 11.0) Post-treatment (12 weeks): 17.0 (SD 11.4) Δ −25.7	SPADI Baseline: 44.3 (SD 11.5) Post-treatment (12 weeks): 14.5 (SD 11.7) Δ − 27.0	Measured with SPADI. A 13-item scale (5 items on pain, 8 on disability). Range 0–130, higher is worse. Pain and disability subscales not reported separately. MID = 18 points [40].
			*Between-group difference in change:* *Δ 1.3; in favor of control (ns)*		
Vallés-Carrascosa et al. (2018) [32]	I: 11 C: 11	I: 0 C: 0	Constant-Murley Score Baseline: 35.0 (22.0; 47.0) Post-treatment (4 weeks): 59.0 (50.0; 68.0) Δ + 24.0	Constant-Murley Score Baseline: 36.0 (22.0; 45.0) Post-treatment (4 weeks): 65.0 (55.0; 69.0) Δ + 29.0	Median (1st;3rd quartile)
			*Between-group difference in change:* *Δ 5; in favor of non-painful eccentric exercise (ns)*		

*DASH* Disabilities of the Arm, Shoulder and Hand questionnaire, *WORC* Western Ontario Rotator Cuff index, *SPADI* Shoulder Pain and Disability Index, *MID* minimal important differences, *ns* not significant

## Abbreviations

CI: Confidence interval
CM score: Constant-Murley score
DASH: Disabilities of the arm, shoulder and hand
IV: Inverse-variance
MD: Mean difference
MID: Minimal important difference
MRI: Magnetic resonance imaging
NPRS: Numerical pain rating scale
OSS: Oxford Shoulder Score
RCTs: Randomised controlled trials
RM: Repetition maximum
SMD: Standardised mean difference
SPADI: Shoulder Pain and Disability Index
VAS: Visual analogue scale
WORC: Western Ontario Rotator Cuff index

## References

[1] Luime JJ, Koes BW, Hendriksen IJ, Burdorf A, Verhagen AP, Miedema HS, Verhaar JA (2004). Prevalence and incidence of shoulder pain in the general population: a systematic review. Scand J Rheumatol.

[2] van der Windt D, Koes B, Boeke A, Deville W, De Jong BA, Bouter L (1996). Shoulder disorders in general practice: prognostic indicators of outcome. Br J Gen Pract.

[3] Struyf F, Nijs J, Mollekens S, Jeurissen I, Truijen S, Mottram S (2013). Scapular-focused treatment in patients with shoulder impingement syndrome: a randomized clinical trial. Clin Rheumatol.

[4] Cools AM, Cambier D, Witvrouw EE (2008). Screening the athlete's shoulder for impingement symptoms: a clinical reasoning algorithm for early detection of shoulder pathology. Br J Sports Med.

[5] Huisstede BM, Miedema HS, Verhagen AP, Koes BW, Verhaar JA (2007). Multidisciplinary consensus on the terminology and classification of complaints of the arm, neck and/or shoulder. Occup Environ Med.

[6] Neer CS (1972). Anterior acromioplasty for the chronic impingement syndrome in the shoulder: a preliminary report. J Bone Joint Surg Am.

[7] Rees JD, Wilson AM, Wolman RL (2006). Current concepts in the management of tendon disorders. Rheumatology (Oxford).

[8] Öhberg L, Lorentzon R, Alfredson H (2004). Eccentric training in patients with chronic Achilles tendinosis: normalised tendon structure and decreased thickness at follow up. Br J Sports Med.

[9] Miller BF, Olesen JL, Hansen M, Døssing S, Crameri RM, Welling RJ (2005). Coordinated collagen and muscle protein synthesis in human patella tendon and quadriceps muscle after exercise. J Physiol.

[10] Iannotti JP (1994). Evaluation of the painful shoulder. J Hand Ther.

[11] Langberg H, Ellingsgaard H, Madsen T, Jansson J, Magnusson SP, Aagaard P (2007). Eccentric rehabilitation exercise increases peritendinous type I collagen synthesis in humans with Achilles tendinosis. Scand J Med Sci Sports.

[12] Mahieu NN, McNair P, Cools A, D'Haen C, Vandermeulen K, Witvrouw E (2008). Effect of eccentric training on the plantar flexor muscle-tendon tissue properties. Med Sci Sports Exerc.

[13] Rutland M, O'Connell D, Brismée J-M, Sizer P, Apte G, O'Connell J (2010). Evidence-supported rehabilitation of patellar tendinopathy. N Am J Sports Phys Ther.

[14] Hanratty CE, McVeigh JG, Kerr DP, Basford JR, Finch MB, Pendleton A (2012). The effectiveness of physiotherapy exercises in subacromial impingement syndrome: a systematic review and meta-analysis. Semin Arthritis Rheum.

[15] Abdulla SY, Southerst D, Côté P, Shearer HM, Sutton D, Randhawa K (2015). Is exercise effective for the management of subacromial impingement syndrome and other soft tissue injuries of the shoulder? A systematic review by the Ontario protocol for traffic injury management (OPTIMa) collaboration. Man Ther.

[16] Kromer TO, Tautenhahn UG, de Bie RA, Staal JB, Bastiaenen CH (2009). Effects of physiotherapy in patients with shoulder impingement syndrome: a systematic review of the literature. J Rehabil Med.

[17] Camargo PR, Avila MA, Alburquerque-Sendín F, Asso NA, Hashimoto LH, Salvini TF (2012). Eccentric training for shoulder abductors improves pain, function and isokinetic performance in subjects with shoulder impingement syndrome: a case series. Rev Bras Fisioter.

[18] Bernhardsson S, Klintberg IH, Wendt GK (2011). Evaluation of an exercise concept focusing on eccentric strength training of the rotator cuff for patients with subacromial impingement syndrome. Clin Rehabil.

[19] Jonsson P, Wahlström P, Ohberg L, Alfredson H (2006). Eccentric training in chronic painful impingement syndrome of the shoulder: results of a pilot study. Knee Surg Sports Traumatol Arthrosc.

[20] Green S, Buchbinder R, Hetrick S (2003). Physiotherapy interventions for shoulder pain. Cochrane Database Syst Rev.

[21] Kelly SM, Wrightson PA, Meads CA (2010). Clinical outcomes of exercise in the management of subacromial impingement syndrome: a systematic review. Clin Rehabil.

[22] Ortega-Castillo M, Medina-Porqueres I (2016). Effectiveness of the eccentric exercise therapy in physically active adults with symptomatic shoulder impingement or lateral epicondylar tendinopathy: a systematic review. J Sci Med Sport.

[23] Valier AR, Averett RS, Anderson BE, Welch Bacon CE (2016). The impact of adding an eccentric-exercise component to the rehabilitation program of patients with shoulder impingement: a critically appraised topic. J Sport Rehabil.

[24] Dervey E, Marshall S, Rouse S (2014). Eccentric exercise therapy in the treatment of subacromial impingement syndrome: a critical review. Int J Ther Rehabil.

[25] Maenhout AG, Mahieu NN, De Muynck M, De Wilde LF, Cools AM (2013). Does adding heavy load eccentric training to rehabilitation of patients with unilateral subacromial impingement result in better outcome? A randomized, clinical trial. Knee Surg Sports Traumatol Arthrosc.

[26] Holmgren T, Björnsson Hallgren H, Öberg B, Adolfsson L, Johansson K (2012). Effect of specific exercise strategy on need for surgery in patients with subacromial impingement syndrome: randomised controlled study. BMJ..

[27] Moher D, Liberati A, Tetzlaff J, Altman DG (2009). PRISMA Group. Preferred reporting items for systematic reviews and meta-analyses: the PRISMA statement. PLoS Med.

[28] PEDro physiotherapy evidence database. Sydney, Australia. [cited 28 May 2018]. Available from: https://www.pedro.org.au.

[29] Maher CG, Sherrington C, Herbert RD, Moseley AM, Elkins M (2003). Reliability of the PEDro scale for rating quality of randomized controlled trials. Phys Ther.

[30] Macedo LG, Elkins MR, Maher CG, Moseley AM, Herbert RD, Sherrington C (2010). There was evidence of convergent and construct validity of physiotherapy evidence database quality scale for physiotherapy trials. J Clin Epidemiol.

[31] Furlan AD, Pennick V, Bombardier C, van Tulder M (2009). 2009 updated method guidelines for systematic reviews in the Cochrane Back review group. Spine (Phila Pa 1976).

[32] Vallés-Carrascosa E, Gallego-Izquierdo T, Jiménez-Rejano JJ, Plaza-Manzano G, Pecos-Martín D, Hita-Contreras F (2018). Pain, motion and function comparison of two exercise protocols for the rotator cuff and scapular stabilizers in patients with subacromial syndrome. J Hand Ther.

[33] Greco T, Biondi-Zoccai G, Gemma M, Guérin C, Zangrillo A, Landoni G (2015). How to impute study-specific standard deviations in metaanalyses of skewed continuous endpoints?. World J Meta-Anal.

[34] Hao Q, Devji T, Zeraatkar D, Wang Y, Qasim A, Siemieniuk RAC (2019). Minimal important differences for improvement in shoulder condition patient-reported outcomes: a systematic review to inform a BMJ rapid recommendation. BMJ Open.

[35] Review Manager (RevMan) [Computer program]. Version 5.3. Copenhagen: The NordicCochrane Centre, The Cochrane Collaboration, 2014.

[36] Guyatt GH, Oxman AD, Kunz R, Vist GE, Falck-Ytter Y, Schunemann HJ (2008). What is “quality of evidence” and why is it important to clinicians?. BMJ..

[37] Chaconas EJ, Kolber MJ, Hanney WJ, Daugherty ML, Wilson SH, Sheets C (2017). Shoulder external rotator eccentric training versus general shoulder exercise for subacromial pain syndrome: a randomized controlled trial. Int J Sports Phys Ther.

[38] Blume C, Wang-Price S, Trudelle-Jackson E, Ortiz A (2015). Comparison of eccentric and concentric exercise interventions in adults with subacromial impingement syndrome. Int J Sports Phys Ther..

[39] Dejaco B, Habets B, van Loon C, van Grinsven S, van Cingel R (2017). Eccentric versus conventional exercise therapy in patients with rotator cuff tendinopathy: a randomized, single blinded, clinical trial. Knee Surg Sports Traumatol Arthrosc.

[40] Bateman M, Adams N (2015). A randomised controlled feasibility study investigating the use of eccentric and concentric strengthening exercises in the treatment of rotator cuff tendinopathy. Arthritis Res Ther.

[41] Hallgren HC, Holmgren T, Oberg B, Johansson K, Adolfsson LE (2014). A specific exercise strategy reduced the need for surgery in subacromial pain patients. Br J Sports Med.

[42] Salaffi F, Stancati A, Silvestri CA, Ciapetti A, Grassi W (2004). Minimal clinically important changes in chronic musculoskeletal pain intensity measured on a numerical rating scale. Eur J Pain.

[43] Roy JS, MacDermid JC, Woodhouse LJ (2009). Measuring shoulder function: a systematic review of four questionnaires. Arthritis Rheum.

[44] van Kampen DA, Willems WJ, van Beers LWAH, Castelein RM, Scholtes VAB, Terwee CB (2013). Determination and comparison of the smallest detectable change (SDC) and the minimal important change (MIC) of four-shoulder patient-reported outcome measures (PROMs). J Orthop Surg Res.

[45] Ekeberg OM, Bautz-Holter E, Keller A, Tveitå EK, Juel NG, Brox JI (2010). A questionnaire found disease-specific WORC index is not more responsive than SPADI and OSS in rotator cuff disease. J Clin Epidemiol.

[46] Holmgren T, Oberg B, Adolfsson L, Björnsson Hallgren H, Johansson K (2014). Minimal important changes in the constant-Murley score in patients with subacromial pain. J Shoulder Elb Surg.

[47] Crenshaw AG, Karlsson S, Styf J, Bäcklund T, Fridén J (1995). Knee extension torque and intramuscular pressure of the vastus lateralis muscle during eccentric and concentric activities. Eur J Appl Physiol Occup Physiol.

[48] Westing SH, Cresswell AG, Thorstensson A (1991). Muscle activation during maximal voluntary eccentric and concentric knee extension. Eur J Appl Physiol Occup Physiol.

[49] Peterson M, Butler S, Eriksson M, Svärdsudd K (2014). A randomized controlled trial of eccentric vs. concentric graded exercise in chronic tennis elbow (lateral elbow tendinopathy). Clin Rehabil.

[50] Hyldahl RD, Olson T, Welling T, Groscost L, Parcell AC (2014). Satellite cell activity is differentially affected by contraction mode in human muscle following a work-matched bout of exercise. Front Physiol.

[51] Ojasto T, Häkkinen K (2009). Effects of different accentuated eccentric loads on acute neuromuscular, growth hormone, and blood lactate responses during a hypertrophic protocol. J Strength Cond Res.

[52] Ingwersen KG, Jensen SL, Sørensen L, Jørgensen HR, Christensen R, Søgaard K, et al. Three Months of Progressive High-Load Versus Traditional Low-Load Strength Training Among Patients with Rotator Cuff Tendinopathy: Primary Results from the Double-Blind Randomized Controlled RoCTEx Trial. Orthop J Sports Med. 2017;5(8). 10.1186/1749-799X-8-40. 10.1177/2325967117723292 PMC5576542 28875153

